# Meshed Architecture of Performance as a Model of Situated Cognition

**DOI:** 10.3389/fpsyg.2020.02140

**Published:** 2020-08-21

**Authors:** Shaun Gallagher, Somogy Varga

**Affiliations:** ^1^Philosophy, University of Memphis, Memphis, TN, United States; ^2^School of Liberal Arts, University of Wollongong, Wollongong, NSW, Australia; ^3^Department of Philosophy and the History of Ideas, Aarhus University, Aarhus, Denmark

**Keywords:** situated cognition, performance, task dependency, body schema, functional integration, meshed architecture

## Abstract

In this paper, we engage in a reciprocal analysis of situated cognition and the notion of “meshed architecture” as found in performance studies (Christensen et al., 2016). We start with an account of various conceptions of situated cognition using the distinction between functional integration, which characterizes how an agent dynamically organizes to couple with its environment, and task dependency, which specifies various constraints and structures imposed by the environment (see Slors, 2019). We then exploit the concept of a meshed architecture as a model that provides a more focused analysis of situated cognition and performance. Through this analysis, we show how the model of meshed architecture can be enhanced through (1) the involvement of a more complex set of cognitive processes, (2) a form of intrinsic control, (3) the influence of affective factors, and (4) the role of factors external to the performer. The aim of this paper, then, is twofold: first to work out an enhanced conception of the model of meshed architecture by taking into consideration a number of factors that clarify its situated nature, and second, to use this model to provide a richer and more definitive understanding of the meaning of situated cognition. Thus, we argue that this reciprocal analysis gives us a very productive way to think about how various elements come together in skilled action and performance but also a detailed way to characterize situated cognition.

## What’s the Situation?

*Embodied*, *embedded*, *extended*, and *enactive* approaches to cognition comprise a loose-knit group of research endeavors that endorse the view that the organism’s body and parts of its environment actively participate in the execution of cognition. They differ in their views about how mind and world are entangled. For example, some endorse *epistemological inseparability* (i.e., no full understanding of cognitive processes is possible by studying exclusively what is occurring inside the head) while others also endorse *ontological inseparability* (i.e., the realizers of cognitive processes can sometimes include parts of the body and the environment) (Varga, 2019). Most would agree that such approaches can be grouped along these two claims, but some have argued that the distinctions in the literature are muddled (Rowlands, 2010). For example, some maintain that work in situated cognition investigates cognitive extensions (Clark and Wilson, 2009) while others consider extended cognition as a distinct class of situated cognition (Robbins and Aydede, 2009, p. 3).

Situated cognition, however, can be considered a broad umbrella term that covers all of these various approaches. As such, it is multifaceted. We might think of it in terms of how environmental features both constrain and enable our cognitive processes. On the constraint side, we can think of various material and structural features as directing us to a specific set of affordances, not only for our perceptions and actions, but also for our deliberations and imaginings. At the same time, these same affordances are enabling of our actions and cognitive activities. It is possible to think of these relations in terms of extended or distributed cognition. Various instruments allow us to engage in epistemic actions (Kirsh and Maglio, 1994), and for some cognitive tasks, we require the use of such instruments. To do the math, we may need paper, pencil, abacus, or some form of machine. To solve a problem, we may rely not only on such tools but also on other people or team members with whom to interact, as well as on normative practices and institutions (understood as cognitive institutions – see Gallagher, 2013; Slaby and Gallagher, 2015). At the same time, these practices and institutions may define specific tasks and place limitations on how we approach a problem or on our style of problem solving.

Thinking of situated cognition in this way, we can define our cognitive engagements as spanning a range between *functional integration* and *task dependenc*y. Marc Slors (2019) has recently clarified these concepts in his analysis of cognitive institutions. We think they can generalize to situated cognition more broadly. Following Slors, for example, we can distinguish between (1) the extended mind approach which starts from the single agent and explains how institutions extend the agent’s cognition (Clark and Chalmers, 1998) and (2) the distributed cognition or systems-based approach that shows how cognitive systems emerge from the integration of individual agents (Hutchins, 2014). In this context, Slors defines functional integration as “the extent to which the execution of tasks involves coupling with items external to the brain and body” (Slors, 2019, p. 1189). A high degree of functional integration means that the cognitive process is constituted by this coupling such that without the external resource, we would be unable to engage in the particular activity, while low functional integration signifies an *enabling* relation such that the external resource simply facilitates our activity. In contrast, task dependency

is the extent to which the intelligibility of a task depends on a larger whole of coordinated tasks. Task dependency is a notion that is connected with coordination and planning. It is a normative notion in the sense that high task dependency means that tasks play specific roles in the overall organization of a cognitive system or a cultural cognitive ecosystem, roles that can be played properly or improperly (Slors, 2019, p. 1190).

The legal system, for example, understood as a cognitive institution (Gallagher, 2013) is characterized by high task dependency. Accordingly, to understand what an attorney does requires an understanding of how that role is linked to the roles played by other people, such as judges and clerks, as well as to a codified body of laws and customs. What one might accomplish in this system will depend upon the structure of the particular situation that constitutes a social-normative or institutional practice.

Situated cognition, then, can be categorized by varying degrees of functional integration and task dependency (Table 1).

**TABLE 1:** Forms of situated cognition (from Slors, 2019, p. 1191).

	**Low functional integration**	**High functional integration**
Low task dependency	Embedded cognition	Extended cognition
High task dependency	Symbiotic cognition	Distributed cognition

Embedded cognition is defined by a low functional integration with various resources that nonetheless enables the performance of cognitive activities and where such activities are intelligible without reference to the institutional structure (low task dependency). Distributed cognition, in contrast, involves the right coupling between distributed components, such as artifacts or other agents in a highly functionally integrated system that also requires high task dependency such that the action of any one individual cannot be understood without reference to others’ activities. Extended cognition (in Clark and Chalmers’ sense) involves high functional integration. Otto, for example, is tightly coupled with his notebook, which allows an extension of his cognitive processes, even if writing and consulting a notebook are not processes that necessarily depend on the roles or tasks of others to be intelligible. “Symbiotic cognition,” as Slors terms it, is found in cognitive institutions. In symbiotic cognition, characterized by high task dependency, an individual’s cognitive processes acquire meaning only in a matrix of interrelationships with the activities of others but do not require a high degree of functional integration.

Every participant in a symbiotic system profits from whatever the system as a whole offers (e.g., education, justice, social coordination) while contributing only a small part. The tasks, jobs and roles of others in the system co-define and enable one’s own task, but one does *not* have to perform them or even think about them, while nevertheless benefiting from the overall outcome of the system (Slors, 2019, p. 1198).

Although this is a productive analysis, it is an oversimplification to think of cognitive institutions as strictly symbiotic or characterized specifically in terms of high task dependency (see Gallagher et al., 2019; Petracca and Gallagher, 2020). As Slors (2019, p. 1190) rightly indicates, “both functional integration and task-dependency come in degrees,” and it seems right to think that a cognitive institution, or situated cognition more generally, will always involve varying degrees of task dependency and functional integration (also see Slors, 2020). Furthermore, how one understands a system will depend on where in the system one is looking or perhaps from what epistemic perspective one is looking. Specifically, the distinction between agent-centered and systems-based perspectives involves different epistemological perspectives that may serve different research agendas but does not necessarily define particular institutional processes. From a systems perspective, a system may involve high task dependency. But from an agent-centered perspective, one may see a significant degree of functional integration that defines that agent’s work.

To motivate our strategy of looking at performance studies to provide some detail in this regard, consider that the notion of task dependency, where action may be defined by a particular role in the performance, is clearly relevant to different types of performance. Of course, task dependency will vary across different types of performance, but different tasks or roles, performed by specific participants, may still be clearly defined, for example, when one is playing football, dancing or acting on stage, or playing a concert in the Sydney Opera House or in the local pub. Specifically, one will find variations in the proportion of functional integration versus task dependency. In a standard tonal jazz performance, for example, task dependency may take the lead. There is a structure to the performance – first, playing the “head,” a statement of the main melodic line; then solos where each performer takes turns following rules concerning harmonic and relatively consistent chord changes; and then, after each performer has taken one or two choruses, the group plays the outro, to end the piece. If one is performing a solo improvisation, without an ensemble, team, or musical group, then task dependency may approach zero, and functional integration may be everything. The latter is a different kind of situation from performing with others; but in each case, the performance and the cognition that goes with it are situated within some variable proportion of functional integration and task dependency.

Functional integration defines how individual agents engage with the various elements of the system and, in so doing, enact the system, which loops back to define performance in specific tasks. An explanation that simply highlights the distinction between functional integration and task dependency, however, remains a somewhat abstract account of situated cognition and is not sufficient to account for how situated agents actually couple to environments or how tasks that are institutionally or more broadly socially, culturally, or normatively defined actually shape that environment. At best, it is an initial specification that requires a more detailed account of how it all works. That is, even if an analysis in terms of functional integration and task dependency provides a productive way to categorize different conceptions of situated cognition, it does not explain precisely how an agent functionally couples with the environment or enters into task-related processes. For example, an adequate concept of functional integration needs to include more than just an account of organism–environment coupling; it also needs to explain how the agentive organism itself is integrated so as to facilitate this coupling.

In the remainder of the paper, we want to provide an account of what it is to be a situated agent engaged in some task or performance that involves varying degrees of task dependency and functional integration. To do this, we turn to the model of a meshed architecture developed in performance studies. We propose that by going into some detail about this model, we can flesh out some of the important aspects of situated cognition. In this respect, we argue that there can be a reciprocal or mutual enlightenment between studies of performance and the theory of situated cognition.

## Meshed Architecture in Performance

As long as an agent is not simply a cog in the machine (an indifferent functional part of the system), one can think of her as a skilled performer or as someone who practices some degree of skilled activity. As we will see, this is one way to characterize functional integration, and it involves something more than simply fulfilling a predefined task in an automatic way, although from a systems perspective, this may sometimes appear to be what is happening. In this respect, we want to rule out the idea of a zero-intelligence agent (see Gode and Sunder, 1993) – that is, an agent who, to perform a task, acts in a purely automatic way and whose performance would involve no cognitive contribution. Functional integration is something more than this and involves a process that is both more complex and more subtle. To make this clear, we turn to a debate in performance theory and the specific model of a meshed architecture to clarify the perspective of a situated agent.

In the area of performance studies, Hubert Dreyfus’ well-known analysis of expertise would come close to the zero-intelligence agent. Dreyfus argued that expert performance involves being mindlessly in the flow, since any form of reflective cognition would be disruptive of performance. He regards subjectivity as a lingering ghost of the mental and denies that there is any awareness in absorbed coping (Dreyfus, 2007, p. 373). On this model, as long as things go smoothly, the agent can be on automatic pilot; there is no need for self-consciousness – the latter is called into action only when the agent detects something going wrong (Dreyfus, 2007, p. 377; see Dreyfus, 2005). At the extreme, this view suggests that expert performance is simply a mindless being in the flow. The elite Sri Lankan cricketer Kumar Sangakkara expresses this view: “Basically in batting, you have to be mindless. You’ve done all the practice, you have your muscle memory and your reflexes are more than quick to deal with any kind of delivery. You’ve got to let your body do all those things by itself without letting your mind take control” (Sadikot, 2014).

In contrast, empirical and phenomenological studies of athletics, dance, theater, and musical performance suggest that performance is not mindless; there is always a cognitive element in performance. Moreover, the cognition involved is always a situated cognition. For example, John Sutton et al. (2011) develop a mindful conception of expert skilled performance. It is not just trained habit that allows an expert player of cricket or baseball to hit a hard fastball (which may be traveling at 140 km/h). In order to hit the ball with precision to a particular location, the batter must draw on current context and the conditions that are relevant to the game. Her performance is “fast enough to be a reflex, yet it is perfectly context-sensitive. This kind of context sensitivity requires some forms of mindedness – [an] interpenetration of thought and action exemplified in open skills” (Sutton et al., 2011, p. 80). The expert batter cannot be on automatic pilot; being on automatic pilot would reduce functional integration to being just one piece of machinery fit to task. Batting skill within the context of a game, for example, involves some mindful strategic sense of where the batter will hit the ball in any particular instance.

Skill is not a matter of bypassing explicit thought, to let habitual actions run entirely on their own, but of building and accessing flexible links between knowing and doing. The forms of thinking and remembering which can, in some circumstances, reach in to animate the subtle kinesthetic mechanisms of skilled performance must themselves be redescribed as active and dynamic (Sutton et al., 2011, p. 95).

Automaticity, therefore, cannot address variability or differences in the agentive situation. Skill and innovative performance require flexibility – the expert batter is aware of the specifics of the situation (including his own skills) and is capable of on-the-fly, explicit, considered awareness which allows for strategic decision making in the flow of performance. This includes elective “target control for some features, such as goal, one or more parameters of execution, like timing, force, a variation in the sequence, and so on” (Christensen et al., 2016, p. 50). In this respect, “expert performers precisely counteract automaticity, because it limits their ability to make specific adjustments on the fly…. Just because skillful action is usually pre-reflective, it does not have to be mindless” (Sutton et al., 2011, p. 95).

To say that functional integration is something more than automaticity in the context of skilled performance, then, motivates several questions. First, what are the cognitive processes involved, and second, how precisely do they “reach in” to the basic body-schematic processes of skilled performance?

Christensen et al. (2016) offer a helpful answer to the second question, developing the concept of a *meshed architecture* to explain the integration of perceptual and cognitive elements with body-schematic motoric processes. On this view, performance is neither fully automatic nor fully cognitive. They develop a hybrid view according to which “cognitive control reduces during skill learning as automatic control comes to play an increasing role, but cognitive control continues to make a substantial positive contribution at advanced levels of skill” (Christensen et al., 2016, p. 41). They propose a *meshed* functioning which involves “a broadly hierarchical division of control responsibilities” along a vertical axis, with top-down cognitive control “focused on strategic aspects of performance and [bottom-up] automatic processes more concerned with implementation” (Christensen et al., 2016, p. 43). Control is mediated, not by explicit inferences, but by “situated awareness,” an awareness that is “constructed” over time with the help of attentional control.

To help us understand how the notion of a meshed architecture can generalize more broadly to situated cognition and contribute to an explanation of functional integration and task dependency discussed above, we propose the following clarifications. First, we suggest that the cognitive processes involved in performance are complex and varied and can include a full register that goes from explicit conscious control to implicit pre-reflective consciousness. Second, we argue that control is not just top-down but can also be intrinsic to bottom-up processes. Third, we argue for the importance of affective factors in modulating intrinsic control features. And fourth, especially in regard to situated cognition, it is even more important to consider that the mesh is complicated by a form of horizontal integration. The horizontal axis of integration includes ecological, social, and cultural/normative factors that extend beyond the performing agent but nonetheless constrain or contribute to performance. By making these clarifications, we hope to provide a more adequate view of how functional integration and task dependency work in situated cognition.

## Complex Cognition

The notion of a meshed architecture has been applied to various types of performance, from athletic performance to the performing arts of acting, dance, and music. Different interpretations of a meshed architecture are possible, however, depending on how we answer the first question about how to understand the cognitive processes involved. Some theorists think of these processes in terms of high-order cognition. For example, in his discussion of theatrical acting, Cohen (2013, p. 33) refers to the actor’s “preparatory thinking as she readies herself for the role, and in-performance thinking, which, in an ideal situation, is ‘aligned’ with the [performer’s] action.” For Cohen, when the actor’s thinking is “properly aligned, her tasks are integrated” (Cohen, 2013, p. 16). This, as Tribble (2016) indicates in her discussion of the meshed architecture, would be a top-down process for Cohen, where low-order processes of embodied coping are modulated by higher-order, reflective cognitive aspects.

Likewise, Montero (2010, 2015) challenges the idea that expert performance is somehow effortless or thoughtless. She argues, in opposition to Dreyfus, that for expert dancers, reflection and body awareness are typically not detrimental to the performance. For Montero (2016, p. 38), optimal performance often coincides with reflective, thoughtful performance, where thoughtful means “self-reflective thinking, planning, predicting, deliberation, attention to or monitoring of … actions, conceptualizing … actions, control, trying, effort, having a sense of the self, and acting for a reason.” Montero (2015, p. 90) pointed to qualitative studies in athletics where a more detailed type of conscious monitoring improves performance (also see Shusterman, 2008 for a similar account).

One could think of this as a type of vertical alignment between higher-order cognitive processes and lower-order motor control processes, with different degrees of integration between the higher- and lower-order processes. This is similar to what Christensen et al. (2016, p. 43) have in mind as they describe the mesh as a combination of cognitive (control-related) and automatic processes: thus, “controlled and automatic processes are closely integrated in skilled action, and … cognitive control directly influences motor execution in many cases.” This divides the vertical into two poles: cognitive at the top, descending to do its job in a “smooth,” “adaptive,” or “effortful” fashion (Christensen et al., 2016, p. 52), and automatic bodily processes at the bottom.

It is possible, however, that there are different degrees of vertical integration in the meshed architecture. Again, this goes back to how one answers the first question about the nature of the cognitive processes involved. The answer shifts between a phenomenology that involves a reflective monitoring and one that involves a more minimal pre-reflective awareness. For phenomenologists, pre-reflective self-awareness does not take the body as an intentional object; it rather involves a “performative awareness … that provides a sense that one is moving or doing something, not in terms that are explicitly about body parts, but in terms closer to the goal of the action” (Gallagher, 2005, p. 73). Legrand (2007, p. 512) described this self-awareness in the context of dance performance: “while dancing [a dancer] is intensively attending to [his body]. But he is not attending to it reflectively as an object. Rather, his [prereflective] awareness of his body as subject is heightened” (see Legrand and Ravn, 2009). The expert dancer keeps this awareness “at the front” of his experience without turning his action or his body into an explicit intentional object (Legrand, 2007, p. 512).

In these various accounts, it seems that what Christensen et al. (2016) call situated awareness can be a matter of degree, ranging from thoughtful, reflective consciousness to a thin performative pre-reflective awareness, with different gradations in between, allowing for such variations as selective target control, conscious monitoring, a sense of one’s rightly configured body, performative awareness, and pre-reflective awareness. The phenomenology of performance may thus be complex and varied. Performers are able to shift across a full register, from explicit conscious control to implicit pre-reflective consciousness and to spontaneous body-schematic processes, adjusting their attunement to changing conditions through improvisation.

## Intrinsic Control

One important question for clarifying the notion of functional integration, as we indicated above, is whether we should consider body-schematic processes as fully automatic. Christensen et al. (2016) mentioned this issue with reference to Fitts and Posner (1967, p. 14) who thought that component processes may automate and Jonides et al. (1985) and Logan (1985) who argued that motor control processes overall do not automate. Christensen, Sutton, and McIlwain seemed to treat body-schematic processes as fully automatic and therefore in need of cognitive control in the performance situation (see Stanley, 2011 for a similar view).

Evidence from kinematics, however, suggests that body-schematic processes are not fully automatic and instead are situation specific, adaptive, and highly dynamical, which facilitates movement in particular situations and for specific intentions. A particular action intention or goal requires the alignment of lots of moving parts in a controlled integration, across varying timescales, many of which are too fast for conscious control. In this respect, however, body-schematic processes are neither fully automatic (blindly doing the same thing in each circumstance and therefore requiring propositional guidance) nor “perfectly general” (Stanley, 2011) but rather include a specificity that depends on an “enormous number (which often reaches three figures) of degrees of freedom” (Bernstein, 1984), as well as a complex temporal organization involving anticipatory processes across skeletal geometry, kinematic phase constraints, muscular geometry, and the dynamics that characterize the relationship between kinematics and geometry (Berthoz, 2000; Gallagher and Aguda, 2020). These complex processes come to align with a particular intention, *not automatically* but in heedful attunement with the particularities of the situation.

Functional integration within such constraints may tune motoric organization to the point where it can become habitual – which may mean *close to* automatic, or automatic in some aspects, but not fully automatic. Merleau-Ponty (2012, p. 143) argued that a habit is formed when the body “acquires the power of responding with a certain type of solution to a certain form of situation.” Habit involves an intelligent response, characterized by openness and adaptivity, so that in familiar or unfamiliar situations, the body learns to cope. As such, intelligence is built into the movement. Instead of blind automatic repetition, habit is intrinsically intelligent. John Dewey likewise distinguished between intelligent and routine habit.

Repetition [i.e., automaticity] is in no sense the essence of habit.… The essence of habit is an acquired predisposition to *ways* or modes of response…. Habit means special sensitiveness or accessibility to certain classes of stimuli, standing predilections and aversions, rather than bare recurrence of specific acts (Dewey, 1922, p. 42).

On this view, performance involves not simply a top-down integration of cognition constraining or guiding automatic processes. Motoric processes in expert performance are already context sensitive, anchored in the situation, but at the same time smart, open, and adaptive, such that they elicit or shape or enable the cognitive elements required for performance. Not only are such cognitive elements, as already noted, complex, including heedful and goal-oriented forms of (attentive, perceptual) consciousness, selective target control, conscious monitoring of action, a sense of one’s rightly configured body, and/or a heightened pre-reflective awareness, but also in such cases, mindfulness is not simply imported from the top; it is already built into the bottom, and again in some cases, such habitual processes may be what guides any need for more reflective cognitive processes. We can call this a kind of intrinsic control.

To summarize, for Christensen et al. (2016), the meshing involves a vertical axis of top-down cognitive control that introduces guidance for bodily processes that remain, at the bottom, automatic. This particular conception of the hybrid mesh, as Høffding and Satne (2019) suggested, is similar to the hybrid car that combines two different elements, battery and fuel. In contrast, they suggested that the mesh may be closer to a fusion – more like an okapi (a unique animal born of zebra and giraffe) than a hybrid car that alternates between the current of automaticity and the fuel of high-octane cognition^1^. An okapi-style mesh, on our view, has a more integrated structure. Practiced and habitual movements (which are neither straightforwardly nor fully nor necessarily automatic) play an important role in an intrinsic control process. Variations in heedful and targeted (attentive, perceptual) awareness are constrained and enabled by a consolidation of fine, detailed motor control (body-schematic) processes, which are not perfectly general or automatic but attuned to the specifics of the situation.

## Affect and Horizontal Meshing

We can get a better idea of what other factors contribute to the meshed architecture by considering an example of musical performance. Simon Høffding’s (2019) study of the Danish String Quartet provided some evidence that the meshed architecture involves both a complex vertical and horizontal integration. Thus, for example, concerning the vertical axis, we find considerations, similar to those above, about the role of thoughtful performance ranging from explicit reflective thinking to pre-reflective awareness and, in some cases, a form of deep absorption where close to automatic processes of the body schema do most of the work. Along this line, Høffding and Satne (2019) interpreted the notion of a meshed architecture as focused on mediating processes rather than the all-or-nothing “automatic” versus “full cognitive” control (also see Salice et al., 2017).

The other factors that Høffding’s analysis considers, in addition to the reciprocal vertical integration of cognition and body-schematic attunement, include affect but also the music itself and intersubjectivity, i.e., the other players. We conceive of the latter two factors as clearly on a horizontal axis which reaches aspects that most theorists would consider as constitutive of the agent’s situation. Affectivity, however, is central and may define the vertical–horizontal intersection.

Affect in the broadest sense includes emotion processes but also more general and basic bodily states such as hunger, fatigue, and pain. Affect, or what Michelle Maiese (2018) calls “affective framing,” shapes our ability to cope with the surrounding world (Ratcliffe, 2012; Colombetti, 2014) and, along with skills and habits, introduces possible modulations of functional integration with that world. Affect may work differently in different types of skilled actions, for example, in various athletic performances and in the different performing arts. The important differences may have to do with the way that affective factors are integrated with motoric/agentive factors – the kinetic and kinesthetic feelings associated with body-schematic processes and how all of these processes functionally integrate with environmental constraints and affordances. Affect/emotion may involve expressive movement, as in dance – movement that is like gesture and language but nonetheless depends on motor control – although it goes beyond simple motor control or instrumental action. There are different mixes or integrations of expressive and instrumental movements in the different contexts of performance – in athletics, dance, or musical performance, for example.

The body schema does not work independently to deliver technically proficient movement, to which an expressive style is then added as something motivated by specific and perhaps occasion-relative emotions. Affective processes directly shape body-schematic processes, slowing them or speeding them or leading them to a certain initial posture that may influence performance or change how agents are functionally situated. Accordingly, affect modulates functional integration. Affect and body-schematic processes are part of the vertical mesh in expert performance – but they also allow for an integration attuned to targets and environmental features in the performance situation.

In the context of musical performance, once we start to think about the music itself and the other performers, for example, we come to an enriched conception of the meshed architecture that incorporates a form of horizontal integration. In this respect, ecological, normative, cultural, and intersubjective aspects of the physical and social environments, including physical and social affordances, play a role and contribute to task-dependent structures in performance. For example, in Høffding’s analysis, the musical instruments, the performance space, and the music itself shape the musical performance. The style of music, whether one is playing from a score, or whether improvisation occurs – these factors establish different roles and tasks and specify different possible dynamics in performance. All of this, in line with embodied-enactive conceptions of affordance-based action and cognition, as well as ecological psychology’s conception of resonance, helps to show that what makes performance what it *is* is not entirely inside the performer, whether she be musician, dancer, athlete, or expert in everyday affairs. For example, the individual performer affectively resonates with and through the music. Playing the musical notes initiates a resonance between the sounds one creates and the musical sounds in the environment made by other musicians.

This resonance may be driven by (1) consciously anticipated, and sometimes planned, notes and/or (2) feedback from awareness of the sounds that are actually created during performance. On one hand, as the music unfolds, the performance environment is constituted as a niche of musical affordances. The sounds that a musician produces could thus successfully or unsuccessfully resonate with the affordances in the environment. On the other hand, anticipatory processes and any short-term planning involved while playing suggest intraorganism resonant loops constantly underlying the performance (Ryan and Gallagher, 2020).

The combination of these respective elements is the mesh between anticipatory control, practiced/skilled bodily movements, and the affordances presented by the music and the environment more generally.

As one engages in a particular performance, one’s agency (or sense of agency) may be modulated (causally influenced) by affect but also by the quality and quantity of affordances available. When, for example, the performer “can ‘feel’ that her motor system has the right configuration” (Christensen et al., 2016), this configuration is just the right one to mesh with the specifics of the performer’s physical and social environments. Høffding (2019, p. 244) called this “interkinaesthetic affectivity” (see Salice et al., 2017; Høffding and Satne, 2019). Neither body-schematic processes nor affective processes are isolated from the agent’s environment; rather, they are attuned to both stabilities and variations in environmental factors, including other agents. The performance and the cognition involved in it are situated, i.e., functionally integrated with the environment. Likewise, environmental factors, including music and interpersonal relations, can facilitate emotion regulation or regulation of affect more generally (Krueger, 2014, 2019).

The environment where performance takes place is not only physically but also socially, culturally, and normatively defined. Performance in a concert hall or in a church may be quite different from performance in a stadium or a pub or in the open air. That we are playing music with others, and who those others are, how skilled they are, and how long we have interacted with them – all of these factors can impact performance (Clarke et al., 2015). If one is playing music with others, there will be an intersubjective and affective resonance between an individual’s performance and the performance of other musicians. This may be mediated by the music itself, by conscious, non-conscious, and/or non-verbal perceptual cues in the others’ embodied performance (see Høffding, 2019; Høffding and Satne, 2019). In some cases, there may also be resonance between the musical group and the audience. These different situations do not entail autonomous high-church cognitive processes – as if what is required is a thinking or reflective contemplation. The performer does not think: “I’m in the concert hall playing with my quartet; therefore I should play in this style.” It is rather that the concert hall and the people I am making music with elicit a specific feeling and style.

In some cases, a strong functional integration can be found in a form of musical joint attention, a shared sense of the music, a kind of entrainment and sensorimotor synchronization with the other players that produces a joint musical experience that approaches Merleau-Ponty’s notion of intercorporeity.

The intercorporeal inclusion of the other musician can be said to alter and expand the sense of agency, such that I no longer primarily attend egoically to my agency, my movements, my interpretation but see the entire setting, music, body, instrument, and even fellow musicians as one large agent. This is an affective and bodily we-intentionality: a musical intercorporeity or musical interkinesthetic affectivity (Høffding, 2019, p. 244).

The meshing of the horizontal and vertical axes may also involve “joint body schemas” in practices that have been shown to extend an individual’s peripersonal space to include the other person, evidenced in changes to neuronal and behavioral processes (Soliman and Glenberg, 2014). As Soliman and Glenberg showed, these body-schematic effects are not simply modulated top-down by cultural practices, but rather, such social and cultural factors are incorporated into body-schematic processes which, in turn, express them in motoric performance. The situation of performance thus involves distributed and temporally extended processes that include all relevant variables – embodied, ecological, intersubjective/social, and cultural. These are not the accomplishments of narrow processes taking place just in-the-head, or strictly on a vertical axis, but are processes that extend into the world, meshed with the structures of our intercorporeal and material engagements.

Accordingly, there are multiple complex factors that extend on the horizontal axis and that shape the situation in which the agent is embedded. The notion of task dependency, where action may be defined by a particular role in the performance, is clearly relevant at this point, although it varies across different types of performance. Returning to the example of the standard tonal jazz performance mentioned in the first section, we find there clear normative task-dependent constraints that specify performance. Such constraints will vary across some proportion of functional integration and task dependency defining both vertical and horizontal meshing. Accordingly, how the “head,” the solos, and the outro actually play out will in varying degrees depend on not just the vertical mesh of cognitive and intrinsic body-schematic control for each individual player but also on our interactions with co-players, on the music itself, and on affective modulations that may permeate the entire situation.

## Conclusion: Performance and Situated Cognition as Mutually Enlightening

We have argued that the notion of a meshed architecture can generalize beyond performance studies and contribute more broadly to an understanding of situated cognition. Once we understand that performance is not mindless, the model of a meshed architecture allows us to specify not only how cognition plays a role in performance but also how other factors situate performance. In regard to this model, we proposed four clarifications: (1) that the cognitive processes involved in performance are complex and varied and can include a full register that goes from explicit conscious control to implicit pre-reflective consciousness; (2) that control is not just top-down but can also be intrinsic to bottom-up processes; (3) that the mesh is complicated by the horizontal integration of ecological, social, and cultural/normative factors that extend beyond the performing agent but nonetheless constrain or contribute to performance; and (4) that affective factors are central for both modulating intrinsic control features and integrating the vertical and horizontal axes of the mesh. By making these clarifications, we have provided a more adequate view of how functional integration and task dependency work in situated cognition.

Accordingly, we think that the meshed architecture model of performance provides a way to explicate various processes involved in situated cognition and helps to make the concepts of functional integration (the coupling of agent and world) and task dependency (most typically defined by social, cultural, and normative factors on the horizontal axis) less abstract. At the same time, notions of situated cognition that involve functional integration and task dependency help to enrich the conception of a meshed architecture.

## Data Availability Statement

All datasets generated for this study are included in the article/supplementary material, further inquiries can be directed to the corresponding author.

## Author Contributions

All authors listed have made a substantial, direct and intellectual contribution to the work, and approved it for publication.

## Conflict of Interest

The authors declare that the research was conducted in the absence of any commercial or financial relationships that could be construed as a potential conflict of interest.

## Footnotes

1 Christensen and Sutton (2019) seemed to move closer to the okapi model. They relaxed the strong dualism between cognition and automaticity (an either–or arrangement), opting for more hybrid or pluralist (both–and) arrangements: “in which there are multiple levels of control, including lower-level, fast perception-action loops and higher-level loops that integrate more widely and process more abstract information, with the loops functioning in intimate interaction” (Christensen and Sutton, 2019, p. 160).
